# Repetitive and self-injurious behaviors: associations with caudate volume in autism and fragile X syndrome

**DOI:** 10.1186/1866-1955-5-12

**Published:** 2013-05-02

**Authors:** Jason J Wolff, Heather C Hazlett, Amy A Lightbody, Allan L Reiss, Joseph Piven

**Affiliations:** 1 Carolina Institute for Developmental Disabilities, University of North Carolina at Chapel Hill School of Medicine, CB# 3367, Chapel Hill, NC, 27599, USA; 2 Department of Psychiatry, University of North Carolina at Chapel Hill School of Medicine, CB# 7160, Chapel Hill, NC, 27599, USA; 3 Center for Interdisciplinary Brain Science Research, Stanford University, 401 Quarry Rd., MC 5795, Stanford, CA, 94305, USA; 4 Department of Psychiatry and Behavioral Sciences, Stanford University, 401 Quarry Rd., MC 5717, Stanford, CA, 94305, USA

**Keywords:** Autism, Caudate, Fragile X syndrome, Repetitive behavior, Self-injurious behavior

## Abstract

**Background:**

Following from previous work suggesting that neurobehavioral features distinguish fragile X and idiopathic variants of autism, we investigated the relationships between four forms of repetitive behavior (stereotypy, self-injury, compulsivity, ritual behavior) and caudate nuclei volume in two groups: boys with fragile X syndrome, a subset of whom met criteria for autism, and a comparison group of boys with idiopathic autism.

**Methods:**

Bilateral caudate nuclei volumes were measured in boys aged 3 to 6 years with fragile X syndrome (*n* = 41), the subset of boys with fragile X syndrome and autism (*n* = 16), and boys with idiopathic autism (*n* = 30). Repetitive behaviors were measured using the Repetitive Behavior Scales-Revised.

**Results:**

For boys with idiopathic autism, left caudate volume was modestly associated with self-injury, while both compulsive and ritual behaviors showed significant positive correlations with bilateral caudate nuclei volumes, replicating previous results. For boys with fragile X syndrome, there was no such association between caudate volume and compulsive behaviors. However, we did identify significant positive correlations between self-injury total scores and number of self-injury topographies with bilateral caudate nuclei volumes.

**Conclusions:**

These findings suggest a specific role for the caudate nucleus in the early pathogenesis of self-injurious behavior associated with both idiopathic autism and fragile X syndrome. Results further indicate that the caudate may be differentially associated with compulsive behavior, highlighting the utility of isolating discrete brain-behavior associations within and between subtypes of autism spectrum disorder.

## Background

Repetitive behaviors are common to many neurodevelopmental disorders, including fragile X syndrome (FXS) and idiopathic autism (iAut). Of these, self-injurious behavior (SIB) is a particularly troubling form of repetitive motor behavior that involves purposeful and repeated patterns of self-inflicted bodily injury without intent of suicide. The behavior is differentially expressed across conditions and individuals, with topographies ranging from minor cases of superficial self-harm to severe forms involving permanent and even life-threatening tissue damage. Self-injury has been reported to occur in as many as 60% of children with FXS [1,2] and 50% of children with iAut [3,4]. While the ontogeny of repetitive motor behavior and SIB in persons with neurodevelopmental disorders is not well characterized, prototypic or early forms of the behavior appear to emerge in early childhood [5].

Despite the significant impact that self-injurious and other repetitive behaviors can have on affected individuals and their families, relatively little is known about underlying neurobiology. Indirect and direct evidence from clinical and preclinical models alike strongly implicate cortico-striato-thalamo-cortical circuitry in the expression of repetitive and stereotyped behaviors including SIB [6]. Numerous preclinical studies have further linked motor stereotypy and SIB with striatal dopaminergic signaling, the dysregulation of which impacts motor execution and related functions central to cortico-striato-thalamo-cortical circuitry [7,8]. Both dopamine deficiency and decreased caudate nuclei (CN) volume have been observed in the striatum of adults with Lesch-Nyhan syndrome, a genetic disorder commonly associated with chronic self-injury [9]. While altered striatal morphology [10-12] and atypical dopaminergic activity [13,14] have similarly been implicated in FXS, little is yet known about the direct relationship (if any) between either the structure or function of the striatum and repetitive behaviors associated with the disorder.

To our knowledge, there are no published neuroimaging data pertaining specifically to SIB associated with either FXS or iAut. However, there is a limited body of work investigating the pathophysiology of other forms of repetitive behavior associated with FXS and iAut using structural brain imaging. Existing studies have largely centered on the dorsal striatum in general and CN in particular. In one study of children and adolescents with FXS, the total volume of the CN was positively associated with two measures of stereotyped motor behavior [15]. CN volume has likewise been associated with various forms of repetitive behaviors in studies of individuals with iAut [16-19], including stereotyped motor behaviors, ritual and compulsive behaviors, and restricted interests and routines. There are, however, some discrepancies regarding the nature of the relationship between the CN and discrete forms of repetitive behavior in iAut with findings inconsistent across studies [19]. Repetitive behavior aside, there is converging evidence suggesting that CN overgrowth is a shared feature of the neural phenotype of both FXS and iAut [10-12,20-22].

We have previously found that in young males with FXS, the CN is highly enlarged relative to boys with iAut, who themselves show significant enlargement relative to boys with typical or delayed development, that is FXS > iAut > DD/TD [10-12,22]. We have separately identified [23] that the behavioral phenotype for preschool-aged boys with FXS and autism (FXS+Aut) is in part characterized by high rates of primarily motoric, or lower-order, forms of repetitive behavior, for example, motor stereotypy and SIB, while boys with iAut are differentiated by significantly elevated compulsive and ritual, or higher-order, repetitive behaviors. Building from these separate brain and behavior findings, our aim in this study was to examine whether differential patterns of brain-behavior associations were evident between these two variants of autism. As an ancillary aim, we were interested in testing our rationale that FXS might serve as a stable model of known etiology against which specific brain-behavior relationships relevant to autism might be mapped, that is, caudate volume and lower-order repetitive behavior (self-injury and motor stereotypy). To this end, we investigated the relationship between bilateral CN volume and repetitive behaviors previously identified as most similar (stereotypy and self-injury) and most dissimilar (compulsive and ritual) in 3 to 6 year old boys with iAut and FXS+Aut [23]. For the purpose of specificity, we also examined our behaviors of interest in relation to a control structure, the amygdala, a region not directly implicated in the neural circuitry believed to underlay repetitive behavior [6].

## Methods

### Subjects

Boys with FXS and iAut were recruited from Stanford University and the University of North Carolina as part of a collaborative imaging study. Inclusion criteria were: (1) complete volumetric MRI data for total gray, white, cerebral spinal fluid (CSF), and bilateral CN; and (2) complete Repetitive Behavior Scales-Revised (RBS-R) data [24]. For the FXS group, full mutation status was confirmed by DNA testing using Southern blotting. The presence of autistic disorder was determined by assessments with the Autism Diagnostic Observations Schedule-Generic (ADOS-G) [25] and Autism Diagnostic Interview-Revised (ADI-R) [26] . Exclusion criteria for the parent study included: (1) history of central nervous system (CNS) injury (for example, cerebral palsy, peri- or post-natal trauma, drug or alcohol exposure); (2) tuberous sclerosis; (3) premature birth (<34 weeks); (4) low birth weight (<2000 g); (5) seizures; and (6) significant motor or sensory impairment (for example, visual impairment, deafness). Children in the iAut group were screened and excluded for evidence of *FMR1* mutations. Given these criteria, the study sample included 41 boys with FXS, including a subgroup of 16 boys with FXS and autism (FXS+Aut), and 30 boys with iAut. Approval of all study procedures was obtained from the University of North Carolina and Stanford University Institutional Review Boards. Informed written consent was obtained from parents or legal guardians for each participant. A detailed description of recruitment and general study procedures may be found in Hazlett *et al*. [10].

### Clinical measures

The ADOS-G is a semi-structured and standardized assessment designed to elicit behaviors associated with autism, while the ADI-R is a structured, standardized parent interview. Children classified as FXS+Aut or iAut met combined ADOS-G and ADI-R criteria for autism. The ADOS-G and ADI-R were administered by trained clinicians and reliability established between clinical sites. Repetitive behaviors were assessed using the RBS-R [24], an independently validated parent report used to characterize discrete forms of repetitive behavior dimensionally [27]. The RBS-R comprises six subscales reflecting varieties of repetitive behavior, including stereotyped motor behavior, SIB, compulsive behavior, ritualistic behavior, sameness behavior, and restricted behavior. Each subscale yields a total score and score for number of items endorsed, or topographies of behavior. The Mullen Scales of Early Learning were administered to all participants to derive ratio IQ scores and characterize general intellectual level [28].

### MRI acquisition

Participants were scanned on identical 1.5 Tesla GE Signa scanners at either Duke-UNC Brain Imaging and Analysis Center (BIAC) or Stanford-Lucile Packard Children’s Hospital. Image acquisition parameters included: (1) coronal T1 IR Prepared: T1 300 ms, TR 12 ms, TE 5 ms, 20° flip angle, 1.5 mm thickness with 1 NEX, 20 cm FOV, 256 × 192 matrix; and (2) coronal PD/T2 2D dual FSE, TR 7200 ms, TE 17/75 ms, at 3.0 mm thickness with 1 NEX, 20 cm FOV, 256 × 160 matrix. Localizer and phantom scans were used to maintain scanner reliability between sites. Participants were scanned with sedation under the supervision of a pediatric anesthesiologist. Physiological monitoring took place continuously throughout scan and recovery time. All scans were screened for clinical abnormalities by a pediatric neuroradiologist.

### Image processing

Image processing has been described previously [10,12]. Briefly, segmentation and subsequent measurement of brain images was initiated through an automated pipeline, which aligned MRI scan data for each subject using a probabilistic spatial prior template (atlas) through a linear, affine transformation. This step included bias estimation, inhomogeneity correction, and nonbrain stripping procedures. Gray, white, and CSF tissues were segmented for each subject, with intracranial volume (ICV) calculated as the combined total of these three volumes [29,30]. Brain volume segmentation was performed by trained technicians at the UNC image analysis lab.

The bilateral CN was measured using a standardized tracing protocol on ACPC aligned high-resolution T1 images using a semi-automated 3D segmentation tool (IRIS/SNAP) [31,32]. A similar protocol was used to parcellate the bilateral amygdala using high-resolution T1 images aligned to the longitudinal axis of the hippocampus. The IRIS/SNAP tool automatically identifies tissue boundaries and produces segmentation labels, which can be inspected and edited as necessary. The method applies user-defined threshold windows, initialization, and region-growing parameters, and provides substantially less bias than a fully manual tracing protocol. Automated segmentations for the caudate were manually inspected and edited to exclude the nucleus accumbens. Reliability was established by two independent raters using a set of 15 scan images consisting of five individual images presented randomly three times. Intra- and inter-rater reliability for caudate nucleus tracing was *r* = 0.97 and *r =* 0.96, respectively. For the amygdala, intra--rater reliability was *r* = 0.90, and inter-rater reliability was *r* = 0.78, with final tracings performed by a single rater. Using this processing pipeline, amygdala parcellations were produced for all but two subjects (one each from the FXS and iAut groups), for whom segmentations could not be defined owing to insufficient image quality.

### Data analysis

Age at MRI scan and IQ scores between iAut and FXS groups were tested by independent samples Student’s *t* test. To limit the number of tests performed and avoid reproducing existing results, means and standard deviations for brain volumes and overall repetitive behaviors and subscale scores were produced but not statistically compared between groups.

The primary aim of this study stemmed from the *a-priori* hypothesis that differential patterns of brain-behavior associations would be evident between fragile X and idiopathic variants of autism, based on previous work identifying group-level differences in CN volumes [10,12,22] and patterns of repetitive behavior [23]. We focused our analysis on associations between bilateral caudate volumes and two forms of repetitive behavior previously identified as *most congruent* between groups (stereotypy and self-injury) and two forms of repetitive behavior identified as *least congruent* (compulsive and ritual) between groups.

Subject data for measures of bilateral caudate and repetitive behavior were inspected for normality and potential outliers. For all groups, volumetric measures for the caudate and amygdale were found to approximate a normal distribution, based on Shapiro-Wilk tests. The majority of repetitive behavior measures across groups, however, did not satisfy the assumption of normality. Because data for most RBS-R subscales were skewed, and because this measure is ordinal in nature, nonparametric correlations (Spearman’s) were used for subsequent correlation analyses involving RBS-R measures. To identify whether potential third variables (age, IQ) were associated with either repetitive behavior measures or caudate volumes, a preliminary bivariate correlation analysis was performed. There were significant correlations between IQ and both brain imaging measures and RBS-R subscale scores. Age was not significantly associated with these variables. For the primary analysis, partial nonparametric correlations between bilateral caudate volumes and repetitive behavior measures were produced for FXS and iAut groups controlling for IQ and ICV. The ICV was included to adjust for the effect of overall brain size on CN volumes. For the control analyses, we used an identical approach, substituting the bilateral amygdala for the caudate.

## Results

There were no significant differences between groups in age, (*t* (70) = -0.24, *P* = 0.81). Consistent with previous publications on this sample, Mullen IQ scores were significantly higher among boys with iAut, (*t* (70) = 2.5, *P* = 0.03). All descriptive data, including those for MRI and RBS-R measures, are presented in Table 1.

**Table 1:** Sample characteristics

	**FXS (n = 41)**		**FXS+Aut (n = 16)**		**iAut (n = 30)**	
	**Mean**	**Standard deviation**	**Mean**	**Standard deviation**	**Mean**	**Standard deviation**
Age (years)	4.6	0.8	4.8	0.8	4.7	0.7
IQ^a^	55.7	16.6	46.3	14.1	70.5	32.1
RBS-R						
Overall score	20.5	14.5	27.1	17.0	26.9	13.7
Stereotyped	5.7	3.4	7.4	3.5	5.9	3.7
Self-injurious	2.1	2.4	2.6	2.7	2.0	2.6
Compulsive	2.3	2.3	3.4	3.2	4.9	3.1
Ritualistic	2.4	2.8	3.4	3.8	4.2	2.9
**Brain volume (cm**^**3**^**)**						
Intracranial volume	1350.4	89.9	1359.7	108.5	1381.1	1452.7
Caudate left	4.6	0.8	4.9	1.0	3.8	0.6
Caudate right	4.8	0.8	4.9	0.9	4.0	0.6
Amygdala left^b^	1.9	0.2	1.9	0.2	2.2	0.3
Amygdala right^b^	1.7	0.2	1.7	0.2	2.1	0.3

^a^ Ratio IQ from Mullen Scales of Early Learning. ^b^ Two subjects (1 FXS, 1 iAut) excluded for amygdala segmentation due to insufficient image quality. RBS-R = Repetitive Behavior Scales, Revised.

Results from the primary analysis, partial correlations of bilateral caudate volumes with repetitive behaviors of interest (RBS-R total scores and number of topographies or items endorsed), controlling for total ICV and IQ, are presented in Table 2. For boys with FXS, left and right caudate volumes were significantly associated with SIB total score: *pr*_*s*_ (37) = 0.35, *P* = 0.037 and *pr*_*s*_ (37) = 0.49, *P* = 0.002, respectively. Left and right caudate volumes were also significantly associated with number of SIB topographies: *pr*_*s*_ (37) = 0.44, *P* = 0.008 and *pr*_*s*_ (37) = 0.55, *P* < 0.001, respectively. For the subgroup of boys with FXS+Aut, SIB total score was significantly correlated with both left (*pr*_*s*_ (12) = 0.62, *P* = 0.018) and right (*pr*_*s*_ (12) = 0.65, *P* = 0.012) caudate volumes. The number of SIB topographies was also significantly associated with left (*pr*_*s*_ (12) = 0.67, *P* = 0.009) and right (*pr*_*s*_ (12) = 0.66, *P* = 0.01) caudate volumes.

**Table 2:** **Partial correlations**^**a **^**between left and right caudate volumes and repetitive behaviors of interest**

	**FXS**		**FXS+Aut**		**iAut**	
	**Caudate left**	**Caudate right**	**Caudate left**	**Caudate right**	**Caudate left**	**Caudate right**
Stereotyped						
Total score	0.01	0.19	0.04	0.10	0.20	0.08
Topographies	0.09	0.22	0.14	0.13	0.17	0.01
Self-injurious						
Total score	0.35*	0.49**	0.62*	0.65*	0.42*	0.35
Topographies	0.44**	0.55***	0.67**	0.66*	0.44*	0.36
Compulsive						
Total score	-0.16	-0.08	-0.16	-0.15	0.44*	0.48*
Topographies	-0.15	-0.08	-0.27	-0.29	0.50**	0.51**
Ritualistic						
Total score	0.22	0.24	0.38	0.43	0.38*	0.47**
Topographies	0.26	0.28	0.37	0.34	0.33	0.38*

^a^Nonparametric (Spearman’s) adjusted for ICV and IQ. * *P* < 0.05; ** *P* < 0.01; *** *P* < 0.001.

Regarding boys with iAut, left caudate volume was significantly associated with both total score (*pr*_*s*_ (27) = 0.42, *P* = 0.02) and number of topographies (*pr*_*s*_ (27) = 0.44, *P* = 0.018) for SIB. Left and right caudate volumes were significantly associated with compulsive behavior total score: *pr*_*s*_ (27) = 0.44, *P* = 0.017 and *pr*_*s*_ (27) = 0.48, *P* = 0.008, respectively. Left and right caudate volumes were also significantly associated with the number of compulsive behavior topographies: *pr*_*s*_ (27) = 0.50, *P* = 0.006 and *pr*_*s*_ (27) = 0.51, *P* = 0.005, respectively. Ritualistic behavior total scores were significantly correlated with both left (*pr*_*s*_ (27) = 0.38, *P* = 0.04) and right (*pr*_*s*_ (27) = 0.47, *P* = 0.01) caudate volumes. Right caudate volume was significantly associated with the number of ritualistic behavior topographies, *pr*_*s*_ (27) = 0.38, *P* = 0.04. Unadjusted results for FXS, FXS+iAut, and iAut groups are presented in Table 3.

**Table 3:** Unadjusted Spearman correlations between left and right caudate volumes and repetitive behaviors of interest

	**FXS**		**FXS+Aut**		**iAut**	
	**Caudate left**	**Caudate right**	**Caudate left**	**Caudate right**	**Caudate left**	**Caudate right**
Stereotyped						
Total score	0.05	0.19	-0.15	-0.16	0.08	-0.02
Topographies	0.11	0.21	-0.02	-0.06	0.16	0.02
Self-injurious						
Total score	0.35*	0.49**	0.64**	0.68**	0.25	0.18
Topographies	0.44**	0.56**	0.70**	0.71**	0.27	0.19
Compulsive						
Total score	-0.12	-0.05	-0.09	-0.09	0.08	0.10
Topographies	-0.11	-0.05	-0.17	-0.19	0.23	**0.**22
Ritualistic						
Total score	0.24	0.28	0.47	0.52*	0.25	0.32
Topographies	0.28	0.30	0.43	0.47	0.16	0.20

* *P* < 0.05; ** *P* < 0.01.

For the control analyses, all partial correlations between bilateral amygdala volumes and repetitive behaviors (total scores and number of topographies) were nonsignificant (*P* > 0.10) for boys with FXS and the FXS+Aut subgroup. All partial correlations were similarly nonsignificant (*P* > 0.10) for the group of boys with iAut.

**Figure 1:**
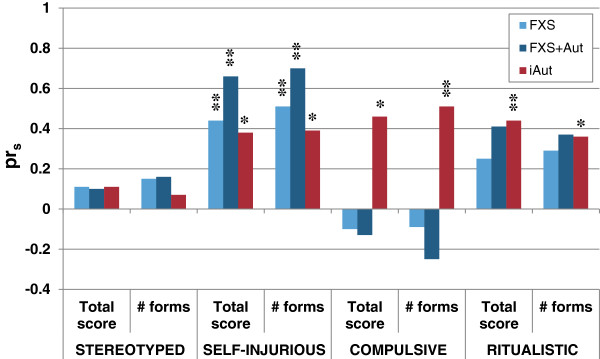
**Relationship of repetitive behaviors to total caudate volume.** Legend: Spearman correlations of total caudate volume to total scores and number of forms endorsed (topographies) for repetitive behaviors of interest as measured by the Repetitive Behavior Scales, Revised, controlling for total brain volume and IQ. FXS: fragile X syndrome; FXS+Aut: fragile X syndrome with autism; iAut: idiopathic autism. * *P* < 0.05; ** *P* < 0.01.

Finally, to better visualize patterns of brain/behavior relationships across groups, we plotted partial correlations for total caudate volume (left + right) with both total scores and number of topographies of repetitive behaviors of interest (Figure 1).

## Discussion

Although repetitive behavior is a cardinal feature of the autism phenotype, it is not a unitary construct, nor is its occurrence unique to autism. There is substantial variability in the form, function, and biological basis of behaviors constituting the repetitive behavior domain. Having previously found that children with FXS+Aut differ from children with iAut in both patterns of repetitive behavior [23] and caudate volumes [10,12,22], we hypothesized that brain-behavior associations would likewise differentiate idiopathic autism from autism associated with an etiologically defined disorder, that is FXS. For boys with both FXS and iAut, we found that overall severity and number of topographies of SIB were positively associated with CN volumes. This pattern was particularly strong among the subgroup of boys with FXS+Aut, who, by definition, are characterized by higher rates of repetitive motor behavior when compared with boys with FXS who do not meet criteria for autism.

Unique to boys with iAut, we found that compulsive and ritual behaviors were significantly correlated with CN volumes consistent with similar studies of older children and adults with the disorder [16,17,19]. While ritual behavior was not significantly associated with caudate volumes in FXS or the FXS+Aut subgroup, these relationships were near the level of significance, suggesting the possibility of a shared mechanism for this specific type of behavior. Interestingly, the same was not true for compulsive behavior, despite presumed overlap in form and function. In general, associations between CN and forms of repetitive behavior were congruent between all boys with FXS regardless of an autism diagnosis, but were in part dissociable from patterns observed in boys with iAut (Figure 1). To ensure specificity of our primary analyses, we also examined the relationship between amygdala volumes and repetitive behaviors. We found no significant correlations between left and right amygdala volumes and any measure of repetitive behavior for boys with FXS, FXS+Aut, or iAut.

Our hypothesis that repetitive motor behaviors would be linked to CN volume in FXS was confirmed with regard to SIB but not stereotypical motor behavior. Although rates of motor stereotypy are significantly elevated in FXS [23,33], we found no relationship between the production of this behavior and CN volume in our sample. The specificity of the self-injury finding lends support to a taxometric rather than a unitary approach to disentangling brain-behavior relationships. That is, while stereotypy and SIB may share common neurobehavioral features, these behaviors might not merely be dimensional expressions of the *same* phenomenon. This argument may extend to distinctions among neural mechanisms underlying similar behavior across genetic disorders. For example, though SIB is common to both FX and Lesch-Nyhan syndromes, the putative neural mechanisms underlying the emergence of functionally similar behaviors might qualitatively differ between these syndromes, as evidenced by inverse patterns of caudate pathology [9-11,20,21]. In this study, SIB was associated with CN volume in boys with iAut in similar fashion to boys with FXS, suggesting some degree of shared pathophysiology. For young children with either FXS or iAut, a larger caudate may indicate an increased risk of developing SIB. This correlation was modest among boys with iAut, however, and statistically significant only for the left CN. The stronger link evident among boys with FXS may be owed to etiological homogeneity. A larger sample of children with iAut might afford the opportunity to define subgroups based on specific neurobehavioral features.

The neural phenotype of FXS, and to a lesser extent iAut, includes significant caudate enlargement irrespective of self-injurious or repetitive behavior [10-12,20-22], In FXS, this morphological feature probably stems from early overgrowth followed by dampened dendritic elimination [34]. Understanding the mechanisms and course of early caudate development in FXS may inform the pathogenesis of SIB. For example, a foundational aberration in caudate structure may confer risk for the development of SIB through altered function or atypical response to feedback [35]. It may be telling that the average age of onset for SIB in children with FXS [2] coincides with the peak and gradual decline of synaptic density in typically developing children [36]. While we can only speculate based on the present findings, it is further possible that both initial and subsequent risk for SIB associated with FXS is tightly linked to atypical developmental processes associated with FMRP (fragile X mental retardation protein), such as reduced synaptic plasticity generally or dysregulated activity involving striatal circuitry specifically [14,34,35]. In this developmental framework, the emergence of SIB may be explained by two possible phenomena. First, SIB risk may be concomitant with extent of striatal malformation stemming from atypical developmental elimination of dendritic spines, particularly in the CN. Second, further increases in striatal volume may result from an additive process associated with altered function stemming from early overgrowth and reciprocally tied to the behavioral performance of SIB.

With regard to this second possibility, findings from preclinical models suggest that significant caudate enlargement can result from chronic dopamine accumulation secondary to D2 receptor antagonism [37]. Persistent D2 receptor antagonism in the dorsal striatum, which includes the caudate, has been associated with chronically elevated nociception (pain sensitivity) [38], a phenomenon that has been observed in persons with SIB using a variety of biobehavioral approaches [39,40]. Similarly, atypical dopaminergic function in the dorsal striatum has been associated with significant elevations in basal stress response [41], evidence of which is seen in numerous studies of human and nonhuman primate studies of SIB [42-44]. Although altered dopaminergic function has been tied to both repetitive self-injury and FXS [7,8,13,14] it remains but one of a number of possible striatal mechanisms underlying motor dysregulation [6]. Volumetric and correlational findings alone cannot speak directly to these issues, and a multimodal, developmental approach involving clinical and preclinical models is necessary to elucidate the complex neurobehavioral processes underlying SIB associated with FXS and iAut [45].

There are several limitations to the present findings. Given our sample size, we were unable to break out subdomains of repetitive behavior by specific form. While the total number of topographies was strongly associated with CN volume, a larger sample is required, to understand whether discrete forms of repetitive and SIBs (for example, self-biting) are differentially linked to brain measures. This study did not account for the influence of environmental variables on behavior, and thus the relationship between brain, behavior, and environment is unknown. Because SIB both shapes, and is shaped by, the environment in which it occurs [46,47], properties such as behavioral function could further inform the brain-behavior associations reported here. Similarly, it would be illuminating to measure to what extent, if any, associated features such as arousal or anxiety moderate the relationship between subtypes of repetitive behavior and brain structure or function [43]. Overall caudate volume is a rather blunt measure of brain anatomy, and the extent to which it informs underlying pathology is limited. It is plausible, for instance, that more nuanced qualities of caudate morphology, beyond overall volume, drive associations with repetitive behavior.

Potential next steps include leveraging multimodal imaging to further examine the striatal neurobiology of repetitive behaviors associated with FXS and iAut. Fruitful approaches might include alternative measures of morphometry, such as shape or surface area, as well as diffusion tensor MRI, which offers the potential to measure structural properties of cortico-striato-thalamo-cortical connectivity. Given that the relationship between the striatum and repetitive behavior is a dynamic one, longitudinal data would provide the opportunity to chart the developmental course of reciprocal brain-behavior change. Such data would likewise allow investigators to explore the utility of striatal neuroimaging measures as a clinically relevant risk marker for SIB. The finding that caudate volume is linked to SIB in both FXS and iAut may provide a common referent from which clinical treatment studies might build. Such work could more closely examine underlying mechanisms with an eye toward well-defined targets, bearing in mind the need to carefully measure discrete classes of behavior rather than blunt outcomes, for example, ‘stereotypy’ and ‘self-injury’ versus ‘repetitive behavior’ or ‘irritability’. Recently, there is exciting promise that an mGlu5 inhibitor could target repetitive behaviors generally and self-injury specifically given demonstrable effects on analog behaviors and dendritic architecture in preclinical studies of FXS and autism [48,49]. In human beings, this promising treatment approach could be used in tandem with behavior analytic strategies aimed at bolstering adaptive replacements for SIB [50]. Finally, replication with larger samples or among individuals with other genetic neurodevelopmental disorders associated with SIB is necessary to confirm and possibly extend the present findings.

## Conclusion

Our present attempt to ‘carve nature at its joints’ through a neurobehavioral approach revealed an association between a specific form of repetitive behavior, SIB, and the morphology of the striatum in boys with both FXS and iAut, while also replicating previous findings pertaining to higher-order repetitive behavior and caudate volume in iAut. As with separate studies of brain and behavior, there would appear to be meaningful areas of both similarity and difference in brain-behavior associations between FXS and iAut [10-12,20-23]. It is worth noting that applying the term ‘autism’ to disparate conditions may lend itself to the overgeneralization of neurobehavioral findings and approaches to treatment [51]. What is true for individuals with iAut, for example, may not be so for individuals with FXS. Alternatively, identifying specific neural mechanisms mediating genotype with discrete features of a behavioral phenotype has the potential to aid attempts to develop targeted and empirically derived early or preventative interventions. For SIB, such efforts are particularly salient given the many challenges associated with treating this often troubling form of repetitive behavior [50].

## Abbreviations

ADI-R: Autism diagnostic interview-revised; ADOS-G: Autism diagnostic observations schedule-generic; BIAC: Brain Imaging and Analysis Center; CN: Caudate nuclei; CNS: Central nervous system; CSF: Cerebral spinal fluid; FMRP: Fragile X mental retardation protein; FXS: Fragile X syndrome; FXS+Aut: FXS and autism; iAut: Idiopathic autism; ICV: Intracranial volume; IQ: Intelligence quotient; RBS-R: Repetitive behavior scales-revised; SIB: Self-injurious behavior

## Competing interests

The authors have no competing interests to declare.

## Authors’ contributions

JP and ALR designed the study. HCH, AAL, JP, and ALR oversaw data collection. JJW and JP analyzed and interpreted the data. JJW drafted the manuscript. All authors contributed to the development of the manuscript.
